# Juvenile Myelomonocytic Leukemia: Molecular Pathogenesis Informs Current Approaches to Therapy and Hematopoietic Cell Transplantation

**DOI:** 10.3389/fped.2014.00025

**Published:** 2014-03-28

**Authors:** Christopher C. Dvorak, Mignon L. Loh

**Affiliations:** ^1^Department of Pediatrics, University of California San Francisco, San Francisco, CA, USA

**Keywords:** juvenile myelomonocytic leukemia, chemotherapy, splenectomy, hematopoietic cell transplantation, immunotherapy, minimal residual disease

## Abstract

Juvenile myelomonocytic leukemia (JMML) is a rare childhood leukemia that has historically been very difficult to confidently diagnose and treat. The majority of patients ultimately require allogeneic hematopoietic cell transplantation (HCT) for cure. Recent advances in the understanding of the pathogenesis of the disease now permit over 90% of patients to be molecularly characterized. Pre-HCT management of patients with JMML is currently symptom-driven. However, evaluation of potential high-risk clinical and molecular features will determine which patients could benefit from pre-HCT chemotherapy and/or local control of splenic disease. Furthermore, new techniques to quantify minimal residual disease burden will determine whether pre-HCT response to chemotherapy is beneficial for long-term disease-free survival. The optimal approach to HCT for JMML is unclear, with high relapse rates regardless of conditioning intensity. An ongoing clinical trial in the Children’s Oncology Group will test if less toxic approaches can be equally effective, thereby shifting the focus to post-HCT immunomanipulation strategies to achieve long-term disease control. Finally, our unraveling of the molecular basis of JMML is beginning to identify possible targets for selective therapeutic interventions, either pre- or post-HCT, an approach which may ultimately provide the best opportunity to improve outcomes for this aggressive disease.

## Introduction

Juvenile myelomonocytic leukemia (JMML) is an uncommon overlap myeloproliferative/myelodysplastic neoplasm that occurs exclusively in young children, with a median age of diagnosis of <2 years (1, 2). Patients frequently present with a high disease burden and severe clinical symptoms, including massive hepatosplenomegaly, pulmonary infiltrates, fevers, infections, and rash (3). With few exceptions, long-term event-free survival (EFS) has only been achieved following hematopoietic cell transplantation (HCT) (4). However, the optimal therapeutic regimen, both before and during the HCT process, has yet to be identified, and large-series published 5-year overall survival (OS) rates range between 52 and 64% (1, 2).

## Establishing the Diagnosis

The clinical picture of JMML can be somewhat non-specific, and until very recently most children with JMML were diagnosed solely upon meeting a certain number of clinical features, such as splenomegaly (present in >90% of cases at diagnosis), a white blood cell count >10,000/μL with circulating immature myeloid forms, an absolute monocyte count (AMC) >1000/μL, increased fetal hemoglobin for age, <20% blasts, and absent BCR/ABL fusion gene (Table 1) (5). Diagnostic confirmation can be enhanced via identification of one of the hallmark features of JMML, such as hypersensitivity of myeloid progenitor cells to the cytokine granulocyte macrophage colony stimulating factor (GM-CSF) in methylcellulose assays (6), which is currently available in only a few CLIA-approved laboratories. GM-CSF-mediated hyperphosphorylation of STAT5 protein in phosphoflow cytometry assays (7, 8) promises to be an exciting technique, but remains currently a research test only.

**Table 1:** **Juvenile myelomonocytic leukemia diagnostic criteria^a^**.

Category 1	Category 2	Category 3
All of the following	At least one of the following	At least two of the following
Splenomegaly^b^ Absolute monocyte count (AMC) >1000/μL Blasts in PB/BM <20% Absence of the t(9;22) *BCR/ABL* fusion gene	Somatic mutation in *RAS* or *PTPN11* Clinical diagnosis of NF1 or *NF1* gene mutation Homozygous mutation in *CBL* Monosomy 7	Circulating myeloid precursors WBC >10,000/μL Increased fetal hemoglobin (Hgb F) for age Clonal cytogenetic abnormality excluding monosomy 7 GM-CSF hypersensitivity

*The diagnosis of JMML is made if a patient meets all of the Category 1 criteria and one of the Category 2 criteria without needing to meet the Category 3 criteria. If there are no Category 2 criteria met, then the Category 3 criteria must be met*.

*^a^Modified from Chan et al. (5)*.

*^b^For 7–10% of patients without splenomegaly, the diagnostic criteria must include all other features in Category 1 and one of the parameters in Category 2, or no features in Category 2 but two features in Category 3*.

Fortunately, deciphering the genetic underpinnings of JMML has facilitated molecular approaches to diagnosis. The association between JMML and certain inherited syndromes advanced understanding of both disorders. For example, the incidence of JMML is increased 200- to 500-fold in neurofibromatosis type 1 (NF1) (9). NF1 encodes neurofibromin, a GTPase activating protein (GAP) for Ras, which negatively regulates Ras output (10). Studies of human JMML specimens and gene-targeted mice show that NF1 acts as a tumor suppressor in myeloproliferative neoplasms and leukemogenesis by inhibiting Ras signaling (11, 12). Noonan syndrome (NS) is characterized by multiple developmental defects, and some patients develop a transient MPN (13, 14). The discovery of germline missense mutations in the PTPN11 gene in ~50% of NS patients (15) proved critical for understanding the associated transient MPN and led to the discovery that somatic PTPN11 mutations are the most common cause of JMML (16, 17). Our recent discovery of homozygous CBL mutations in JMML led to further investigations demonstrating that such lesions are usually heterozygous germline events with somatic loss of heterozygosity in the hematopoietic compartment, similar to what happens in NF1-mutated JMML (18). In addition, somatic NRAS and KRAS mutations occur in approximately equal frequencies in patients with JMML and led to the interesting observation that some patients with NS harbor germline RAS lesions, albeit in alternative codons (19, 20). In total, 85–90% of patients with *de novo* JMML harbor mutations in genes that regulate the Ras pathway: PTPN11 35%, NRAS/KRAS 30%, NF1 loss 10–15% [reviewed in Ref. (21)], homozygous CBL 10% (18, 22). Mice with similar mutations in Nf1, Kras, *Ptpn11*, and *Cbl*, all develop fatal MPN resembling JMML (23–28). Other mutations that have been reported to occur less frequently in JMML include *ASXL1* and *FLT3* (29, 30). Recent whole exome strategies have also identified mutations in *SETBP1* and *JAK3* occurring in up to 16% of JMML patients, and confer a poorer prognosis, though the therapies delivered were variable (31).

Therefore, we can now molecularly confirm the diagnosis of JMML in approximately over 90% of patients. In the remaining patients, other classic JMML features, such as the presence of monosomy 7, can support the diagnosis. In the absence of being definitively diagnosed with JMML, other diseases should be considered, such as Wiskott–Aldrich Syndrome (32) or neonatal CMV infection (33). Unfortunately, due to the rarity of the diagnosis and the relatively recent discovery of pathogenic mutations, it is difficult to determine the prognostic significance of any one of these alterations. Indeed, the majority of published studies have included patient outcomes without molecular confirmation of the disease. The newest generation of trials, however, will include central molecular diagnosis, and will also allow analysis of potential differences between the various genetic subgroups in terms of responses to therapy, as some small studies have suggested that patients with *PTPN11* mutations may have less favorable outcomes (34–36).

The overlap between somatic alterations and germline syndromes has important implications on whether or not to perform a HCT. As stated above, it has long been recognized that patients with NF1 who develop full-blown JMML require HCT for cure. Conversely, patients with germline mutations in *PTPN11* (NS) develop a myeloproliferative disorder that strongly resembles JMML, though nearly all cases appear to spontaneously regress over the first 18 months of life without a need for HCT (14, 37). These patients typically have characteristic findings upon physical examination, and also have codon substitutions that are largely confined to patients with NS, as opposed to the spectrum of somatic lesions that occur in *de novo* JMML. Simultaneous testing of a buccal swab sample can help confirm or rule out NS. Recently, germline mutations in *NRAS* have also been shown to cause some cases of NS (38) and spontaneous regression of JMML (39). Interestingly, it has even been reported that some patients with somatic *NRAS* (especially the *NRAS*^G12S^) (40) and *KRAS* (especially the *KRAS*^G12V^) (41) mutations, may have an indolent course or even spontaneously regress their MPN. Finally, it appears that most patients with JMML due to *CBL* mutations have a germline mutation, either inherited in an autosomal dominant fashion or arising from a spontaneous germline event, with acquired uniparental isodisomy in the leukemic cells. This is often associated with characteristic clinical features, such as café-au-lait spots, juvenile xanthogranulomas, male cryptorchidism, hearing loss, and growth and developmental delay (18). Some patients with *CBL* mutations can have aggressive JMML, while others appear to spontaneously resolve, only to go on later to develop significant, and sometimes fatal, vasculitis (18). This vasculitis does not appear to be seen in patients who undergo HCT, such that we are still recommending HCT for patients with *CBL* mutations who demonstrate an aggressive clinical course, at least until we have a better understanding of why certain patients spontaneously improve.

## Risk-Stratification

To date, there has not been a rigorous development of a risk-stratification system for patients with JMML either at time of diagnosis (to guide potential administration of pre-HCT chemotherapy), or at time of HCT (to guide post-HCT interventions). Various factors have been identified that contribute to poor outcomes (Table 2) (1, 2, 34, 36, 42), though these have not always been replicated between studies. Limited gene-expression profiling and DNA hypermethylation studies have identified signatures that are prognostic of poor outcome and appear independent of genotype (36, 43). As molecular diagnosis improves and new laboratory techniques become available, a major aim of future studies will be the development of a genomics-based risk-stratification system.

**Table 2:** **Juvenile myelomonocytic leukemia high-risk features**.

	EWOG/EBMT	EUROCORD/CIBMTR	UK	Europe	Japan	Conclusion
Number studied (*N*)	100 (1)	110 (2)	67 (42)	44 (36)	71 (34)	
**HOST FACTORS**						
Age at diagnosis (years)	>4	>1.4	>2	>2	>2	Older age is high-risk, best cut-off age has not been defined
Gender	Female	Not seen	Not reported	Not seen	Not seen	Evidence inconclusive
**JMML-SPECIFIC FACTORS**^c^						
Type of mutation	Not seen	Not seen	Not reported	PTPN11 (trend)	PTPN11	PTPN11 may be high-risk
Cytogenetic abnormality	Not seen	Monosomy 7	Not monosomy 7	Monosomy 7	“Abnormal”	Monosomy 7 likely high-risk, evidence conflicting
HgbF at diagnosis	>40%	Not seen	Elevated	Not seen	Not seen	>40% may be high-risk
Platelet count at diagnosis	Not seen	Not seen	<40 × 10^9^/L	Not seen	Not seen	Low platelets may be high-risk
BM blasts at HCT	>20%	Not seen^a^	Not reported	Not seen^b^	Not reported	>20% blasts is probably high-risk
**TREATMENT-RELATED FACTORS**						
Splenectomy	Not seen	Beneficial	Not reported	Not reported	Not reported	Debatable, benefit may be specific to UCBT
Pre-HCT AML-like chemo	Not seen	Beneficial	Not reported	Not reported	Not reported	Debatable, benefit may be specific to UCBT
HLA well-matched donor	Not seen	Beneficial	Not reported	Not seen	Not reported	Debatable, benefit may be specific to UCBT
Serotherapy	Beneficial	Not seen	Not reported	Not reported	Not reported	Debatable, benefit may be specific to non-UCBT

*^a^Patients with >20% blasts at HCT not included*.

*^b^AML-like gene-expression profile is highest-risk*.

*^c^DNA hypermethylation is also high-risk (44)*.

Patients with JMML who transform into AML (currently defined as >20% blasts in bone marrow) generally have dismal outcomes following HCT. As noted above, there was a 0% EFS rate in EWOG-MDS/EBMT trial for patients who entered HCT with >20% blasts (1). Data are relatively lacking on whether these poor outcomes also apply to those who at any point pre-HCT had evidence of AML transformation. However, gene-expression profiling techniques can distinguish a population of “typical” JMML patients (with a 10-year EFS rate of 63%) vs. those with an AML-like pattern (with a 10-year EFS rate of 6%) (36). Given these dismal outcomes, it would be reasonable to consider patients with AML transformed out of JMML for experimental therapies prior to HCT.

## Pre-HCT Treatment

A variety of pre-HCT treatments have been employed to control symptoms of JMML (such as organomegaly and pulmonary problems) as well as theoretically improve outcomes. A common treatment is the use of oral 6-mercaptopurine (50 mg/m^2^/day), sometimes combined with cis-retinoic acid (100 mg/m^2^/day) (45). Low-dose intravenous cytarabine (40 mg/m^2^/day × 5 days) administration has also been used, as has the combination of high-dose cytarabine (2 g/m^2^/day × 5 days) plus fludarabine (30 mg/m^2^/day × 5 days) (46). Pulmonary rebound phenomena can occur after such therapies are delivered and as such, patients need to have careful monitoring during their recovery phase (C. Niemeyer, personal communication). However, to date no study has conclusively demonstrated a benefit of pre-HCT treatment in post-HCT survival outcomes.

## The Role of Pre-HCT Myelosuppressive Chemotherapy

The EWOG-MDS/EBMT trial showed that patients who had no therapy or low-dose chemotherapy compared to high-dose AML-like chemotherapy, had nearly identical rates of 5-year EFS (52 vs. 50%), relapse incidence (35 vs. 38%), and transplant-related mortality (13 vs. 13%) (1). The grouping of patients who got no chemotherapy with those that got low-dose chemotherapy makes it difficult to determine if there is any benefit to low-dose chemotherapy. Furthermore, it is challenging to interpret this data, as the choice of pre-HCT chemotherapy was left to the treating physician’s discretion, and patients with higher disease burdens were likely more intensively pre-treated. A significant number (10%) of patients on this trial had AML transformed out of JMML at time of HCT, and they had a 0% EFS rate. It stands to reason that these were the patients most likely to have previously received AML-like chemotherapy, and therefore may have obscured a potential benefit to intensive chemotherapy in a “standard” patient with JMML.

In the EUROCORD/CIBMTR retrospective study, which specifically excluded patients with >20% blasts at time of umbilical cord blood transplant (UCBT), there seemed to be improved EFS in patients who got AML-like therapy compared to none or low-dose chemotherapy (55 vs. 32%; *P* = 0.048) (2). Interestingly, this improvement in survival did not correlate with post-HCT relapse, but rather with decreased TRM in those who got AML-like chemotherapy (20 vs. 43%; *P* = 0.01). TRM in this study was multi-factorial, including 50% from infections, 17% from organ failure, 17% GVHD, 13% rejection, and 4% hemorrhage. No explanations were offered for this paradoxical finding, but possible hypotheses for a benefit of AML-like chemotherapy on TRM could include: (1) decreased rejection via enhanced immunosuppression and/or smaller spleen sizes; (2) faster count recovery due to smaller spleen sizes resulting in less infections and bleeding; or (3) a component of tumor lysis syndrome during conditioning contributing to organ failure post-HCT. Whether this is an effect specific to UCBT remains to be seen.

Furthermore, these studies only report on whether or not AML-like chemotherapy was utilized, but do not carefully distinguish on whether or not there was a response to that chemotherapy. This is due to the fact that historically measuring responses in patients with JMML has been exceptionally difficult, using only relatively crude measures such as WBC count, BM blast percentage, and spleen size at time of HCT (46). Fortunately, the same technology utilized to detect the diagnostic mutation can now be used to measure disease burden down to minimal amounts of residual disease for patients with mutations in *PTPN11, NRAS, KRAS*, and *CBL* (47). Therefore, future studies will be able to distinguish whether response to chemotherapy vs. simply the use of chemotherapy, prior to HCT impacts on post-HCT relapse and survival.

## The Role of Pre-HCT-Targeted Chemotherapy

Given the current lack of convincing evidence for traditional myelosuppressive chemotherapy pre-HCT, as well as data to support hyperactive Ras/MAPK signaling in JMML, there has been significant attention directed toward developing novel-targeted therapeutics for patients with JMML. In particular, inhibition of signaling through the Ras/MAPK pathway has shown benefit in primary human cells, genetically engineered mouse models of JMML, and induced pluri-potent stem cells (44, 48–50). Together, the data have supported development of early phase clinical trials using MEK inhibitors for JMML. Unfortunately, the therapeutic promise of inhibiting Ras farnesylation has not been borne out in numerous studies of other myeloid malignancies, likely due to an alternative mechanism of activation of Ras via geranylgeranylation. Indeed, outcomes for JMML patients treated with a window of Zarnestra, a farnesyl-transferase inhibitor, was actually worse than patients who did not receive treatment with Zarnestra, though reasons for this are unclear (A. Ward, unpublished data). Recent reports of aberrant DNA methylation contributing to increased risk of relapse (43) has led to a European protocol to employ DNA hypomethylating agents such as 5-azacitidine for therapy. To date, this strategy has only been published in a single patient with a monosomy 7 and a *KRAS* mutation. Over an 18-month timespan, an excellent clinical (resolution of organomegaly, monocytosis, thrombocytopenia, and monosomy 7) and molecular response (disappearance of mutant *KRAS*) was noted in this patient which may have facilitated successful HCT (51).

## The Role of Splenectomy

Pre-transplant splenectomy for patients with JMML is controversial, especially given the long-term risks associated with this procedure (52) and lack of conclusive benefit. The EWOG-MDS/EBMT JMML trial demonstrated that a comparison of children with: (1) a spleen size <5 cm at HCT; or (2) spleen >5 cm at HCT; or (3) post-splenectomy at HCT, showed no statistical benefit in EFS (5-year EFS; 61 vs. 44 vs. 48%, respectively), relapse incidence (5-year RI; 24 vs. 45 vs. 39%, respectively), or treatment-related mortality (5-year TRM; 15 vs. 11 vs. 13%, respectively) (1). However, in the EUROCORD/CIBMTR study, there was a trend toward an EFS benefit for splenectomy (56 vs. 36%; *P* = 0.098) (2). These two findings are not mutually exclusive, as an enlarged spleen at the time of an HCT with smaller cell doses (such as is seen in UCBT) may interfere with a successful engraftment or count recovery, and therefore be associated with more deaths from either TRM or relapse. If pre-HCT chemotherapy fails to adequately reduce splenic size, one theoretical solution would be to provide focal splenic irradiation. There is no published experience with this strategy in patients with JMML, but in a large number of adults with CML, MDS, or myeloproliferative disease, when employed in low doses (median of 900 cGy) immediately prior to HCT (UCBT excluded), splenic irradiation had no benefit (53). However, it remains unclear in patients with JMML whether a higher dose of splenic irradiation (a biologically effective dose of up to 20 cGy can be delivered without permanent splenic dysfunction) (54) at an earlier time from HCT (to allow for effective alteration of the degree of splenic migration) would play a role in splenic shrinkage and hence either relapse prevention or improved engraftment, especially in those receiving UCBTs.

## Approaches to HCT

Outcomes of HCT for JMML remain sub-optimal. For 100 patients transplanted primarily with sibling or adult bone marrow from 1993 to 2002 in 29 European centers, the 5-year EFS was only 52%, with a 5-year OS of 64%. Focusing exclusively on 110 patients who received UCBTs from 1995 to 2010, the 5-year EFS and OS rates were 44 and 52%, demonstrating a lack of significant improvement over time in outcomes other than TRM.

## Conditioning Regimen and the Transplant Process

Only one trial with a large number of JMML patients given a uniform conditioning regimen has been published to date. The EWOG-MDS/EBMT trial utilized myeloablative doses of busulfan (16–20 mg/kg orally over 4 days, a minority with pharmacokinetic-directed targeting to achieve a concentration steady state of 500–800 ng/mL), plus cyclophosphamide (120 mg/kg over 2 days), and melphalan (140 mg/m^2^ once) (BU–CY–MEL). One small report of 10 children given busulfan (560 mg/m^2^ orally over 4 days), plus fludarabine (120 mg/m^2^), and melphalan (180–210 mg/m^2^) demonstrated promising results with 7 patients in remission >2 years since HCT (55). The COG AAML0122 trial employed a phase II window of a farnesyl-transferase inhibitor followed by conventional moderate dose AML-type chemotherapy and cis-retinoic acid. Conditioning utilized cyclophosphamide (120 mg/kg over 2 days) and 1200 cGy total body irradiation (TBI), but compliance was poor with the intended TBI regimen, particularly after the busulfan-based EWOG-MDS/EBMT trial was published. Given the young-age at HCT (2.2–2.5 years) of most patients with JMML (1, 2), it would generally be preferable to avoid TBI-based regimens in order to avoid long-term endocrinologic and neurocognitive damage.

Furthermore, despite this escalation in regimen intensity to something approaching the maximal end of the tolerability spectrum, relapse rates remain very high, and limited progress is being made. The EWOG-MDS/EBMT trial published in 2005 included 100 patients transplanted from 1993 to 2002, with a 5-year OS of 64% (1). The EUROCORD/CIBMTR retrospective study published in 2013 included 110 patients transplanted from 1995 to 2010 (with a median year of 2003), during which time advances in HLA-matching and supportive care techniques significantly improved the TRM rates of patients undergoing unrelated donor HCT (56). However, despite these advances, the 5-year OS for the EUROCORD/CIBMTR retrospective study was only 52% (2).

Therefore, further dose escalations are unlikely to be beneficial, and indeed, the modern trend in allogeneic HCT has been to de-escalate conditioning intensity slightly in order to minimize toxicities. As long as the regimen is still within the realm of myeloablation and successfully achieves donor cell engraftment, it is reasonable to expect that the alloreactive process can be established with long-term control of disease. In fact, there are several retrospective studies that suggest that the relapse incidence for adult patients with AML/MDS is at least comparable (and potentially favors) busulfan plus fludarabine (BU–FLU) over busulfan–cyclophosphamide (BU–CY) (57, 58). The one exception to the intensity-rule is a randomized trial for patients with AML, where TBI was intensified from 1200 to 1575 cGy, and found that relapse rates were lower, with no benefit to survival due to increased toxicity (59). Furthermore, the acute GVHD rates were higher in the 1575-cGy group, suggesting that the relapse protection may not be from a direct anti-leukemic effect, but rather from induction of increased alloreactivity. Similar concerns were recently raised regarding the benefit of melphalan in the conditioning regimen of children with JMML and MDS, where patients who non-randomly received BU–CY alone had less aGVHD of the gastrointestinal tract than those who received BU–CY–MEL, with at least equivalent OS rates (60).

Taking this one step further, another group has non-randomly compared BU–FLU (160 mg/m^2^ over 4 days) to BU–CY–MEL in a mixed group of pediatric patients, and found decreased toxicity with equivalent survival (61). In conjunction with the knowledge that no amount of chemotherapy is curative for the standard patient with JMML, these findings call into question the entire strategy of maximally intense conditioning for JMML. An alternative hypothesis is that the sole purpose of the conditioning regimen is to reliably and safely establish donor cell engraftment and provide a platform for donor alloreactivity to perform the actual disease control. This hypothesis is being prospectively tested in the ongoing COG trial, ASCT1221: a randomized phase II study comparing two different conditioning regimens prior to allogeneic HCT for children with JMML (NCT01824693).

Some other lessons applicable to the HCT process for JMML can be learned via careful evaluation of the published data. First, univariate analysis revealed that TRM was lower (6 vs. 22%; *P* = 0.035) when a male donor was utilized (1). This likely is a reflection of the male predominance of the disease, but highlights the importance of careful donor selection. Furthermore, there was no detriment to longer intervals between diagnosis and HCT for EFS, relapse, or TRM, suggesting that there is time to perform a careful and complete donor search (1). Finally, for UCBT, units with 0–1 HLA disparity resulted in a trend toward improved EFS than those with 2–3 HLA disparities (48 vs. 34%; *P* = 0.07) (2). Many other transplant-related factors had slight trends toward showing a benefit, but failed to achieve statistical significance, possibly due to low numbers. Given the rarity of the disease, trans-continental data-sharing collaborations may be required to gather sufficient numbers to answer some of these questions.

## The Adjunctive Use of Serotherapy and G-CSF

The inclusion of serotherapy (such as anti-thymocyte globulin, ATG) in a conditioning regimen is often controversial due to concerns that potential *in vivo* reduction of alloreactivity may result in increased relapse rates. However, two randomized trials in patients undergoing unrelated donor HCT for various malignancies showed no differences in relapse incidence between those patients who did or did not receive ATG, but did show decreased rates of GVHD (62, 63). The EWOG–EBMT trial reported no difference in 5-year RI in patients with JMML undergoing unrelated donor HCT who did (36%) or did not (33%) receive serotherapy (*P* = NS), though there was a trend toward less non-relapse mortality in the serotherapy group (*P* = 0.08) (1). Most patients who underwent UCBT in the EUROCORD–CIBMTR study did receive serotherapy, with no apparent impact on EFS rates (2). Therefore, at present the current data suggest that the serotherapy should be utilized for those undergoing unrelated adult HCT or UCBT. One consideration, however, would be the optimal timing of the serotherapy for UCBT patients, as some data suggest that earlier administration is beneficial for preventing excessive *in vivo* T cell depletion (61).

Finally, given the intrinsic hypersensitivity of JMML cells to GM-CSF, it has been questioned whether routine administration of G-CSF post-HCT to enhance neutrophil recovery would be associated with an increased risk of relapse. There is no *in vitro* data on whether G-CSF directly stimulates JMML stem cells, nor there is published clinical data on the benefits or risks of G-CSF in patients with JMML. Since the benefit of post-HCT G-CSF for patients with AML or CML is unclear, we generally recommend that it is not given to patients getting BM or PBSC as the stem cell source, especially in patients with monosomy 7-associated disease (by analogy to patients with AML) (22). However, until more data are available, the continued use of G-CSF following UCBT is a reasonable strategy.

## The Graft-vs.-JMML Effect

The EWOG–EBMT trial did not show a definitive benefit of either acute or chronic GVHD for prevention of relapse (1). However, this analysis may have been confounded by the inclusion of patients with very high disease burdens at the time of HCT, in which alloreactive responses may be insufficiently therapeutic. In the EUROCORD–CIBMTR study, grades II–III acute GVHD were associated with a decreased incidence of relapse (*P* = 0.02), while grade IV acute GVHD was not surprisingly associated with high rates of TRM (2). Other small reports have documented instances of a graft-vs.-JMML effect, including demonstrations that immunotherapy-based interventions, either rapid withdrawal of immunosuppression (64, 65), administration of donor lymphocyte infusions (DLIs) (66–70), and/or immunostimulation with interferon-α (71, 72) can result in temporary and rarely long-lasting disappearance of residual/relapsing JMML cells following HCT.

In general, immunomanipulation strategies are most effective in the setting of a low disease burden. In order to accomplish this post-HCT, when a relapse may be just developing, accurate measurement of minimal disease burden via measurement of mutated allelic burden will be necessary (47). Preliminary studies suggest that MRD may be as accurate in peripheral blood as in bone marrow, which would significantly increase the ease of sending frequent measurements (47). Another common approach for post-HCT management of myeloid malignancies is to increase alloreactivity in an attempt to achieve 100% donor chimerism (73). However, it is not clear that 100% donor chimerism is required for all patients with JMML. It has been shown that many patients with *CBL*-mutated JMML are long-term survivors with mixed whole blood chimerism (18), and rare anecdotal cases exist for other molecular subtypes, including *PTPN11* (Christopher C. Dvorak, personal communication). Ultimately, the combination of donor chimerism and molecular MRD measurement will likely prove to be the most accurate method for guiding pre-emptive immunotherapy.

One drawback to aggressive immunomanipulation is the risk of causing GVHD in conjunction with the intended graft-vs.-leukemia (GVL) effect. In the setting of mild to moderate GVHD development, the clinician is faced with trying to determine when to treat and how aggressive to be with treatment intensity, so that not too much of the GVL effect is abrogated. In patients with JMML, in addition to standard corticosteroids, second line agents could be chosen for their potential role in simultaneously controlling the JMML itself. Examples include the mTOR inhibitor sirolimus (74) for acute or chronic GVHD, or dasatinib (75) for chronic GVHD, both which has been shown *in vitro* to directly inhibit growth of some cases of JMML. Another interesting approach described in one case report is to utilize 6-mercaptopurine for control of both acute GVHD and JMML (76).

## Second Transplantation

In the EWOG/EBMT trial, 15 patients underwent second HCT for treatment of relapsed disease, with the original donor used most often and TBI being the most common backbone of the conditioning regimen. A purposeful reduction in GVHD prophylaxis led to a high rate of acute GVHD, but at time of publication almost half of the patients were alive (1). Similar results were seen in a report from Japan where 5 of 11 patients undergoing second HCT were alive at last follow-up (70). Other older reports confirm the general utility of attempting a second (77) or even third HCT (78) for recurrent JMML, while a recent case series of five patients describes a successful cytoreductive approach with high-dose cytarabine (3 g/m^2^ q12 × 6 doses) plus mitoxantrone (10 mg/m^2^/day × 3 days) followed by repeat cell infusion from the original donor (79). Although the numbers are small, when combined, these results are at least comparable, if not superior, to the results of second HCT for recurrent acute leukemia (80).

## Conclusion

Patients with JMML have traditionally been difficult to confidently diagnose, manage, and transplant. Recent work to decipher the genetic underpinnings of the disease has significantly improved our diagnostic capabilities, such that over 90% of patients can now be molecularly characterized. Not only will this allow for mutation-specific risk-stratification in the future, but also will ensure that ongoing and future trials include only patients with confirmed JMML and not some of its mimics (which might influence the findings of past reports). Pre-HCT management of patients with JMML remains primarily symptom-driven, though advances in understanding of high-risk clinical features will determine which patients might benefit from pre-HCT chemotherapy. Furthermore, new techniques to quantify minimal residual disease burden will allow us to finally determine whether pre-HCT response to chemotherapy is beneficial for long-term disease-free survival. HCT for JMML remains problematic, with high relapse rates regardless of conditioning intensity. An ongoing clinical trial in the Children’s Oncology Group may determine that less toxic approaches can be equally effective, thereby shifting the focus to post-HCT immune manipulation strategies for long-term disease control. Finally, our increased understanding of the molecular basis of the disease is beginning to identify possible targets for selective therapeutic interventions, either pre- or post-HCT, which ultimately hold the greatest potential for improving the outcomes of this rare and challenging childhood leukemia.

## Conflict of Interest Statement

The authors declare that the research was conducted in the absence of any commercial or financial relationships that could be construed as a potential conflict of interest.
